# Day 1 neutrophil-to-lymphocyte ratio (NLR) predicts stroke outcome after intravenous thrombolysis and mechanical thrombectomy

**DOI:** 10.3389/fneur.2022.941251

**Published:** 2022-08-09

**Authors:** Siyan Chen, Jianhua Cheng, Qiang Ye, Zusen Ye, Yanlei Zhang, Yuntao Liu, Guiqian Huang, Feichi Chen, Ming Yang, Chuanliu Wang, Tingting Duan, Xiang Liu, Zheng Zhang

**Affiliations:** ^1^Department of Neurology, Wenzhou Medical University Affiliated the First Hospital, Wenzhou, China; ^2^Department of Neurology, The Quzhou Affiliated Hospital of Wenzhou Medical University, Quzhou People's Hospital, Quzhou, China; ^3^Department of Neurology, Wencheng County People Hospital, Wenzhou, China

**Keywords:** neutrophil, lymphocyte, acute ischemic stroke, thrombolysis (tPA), thrombectomy, outcome

## Abstract

**Background:**

The neutrophil-to-lymphocyte ratio (NLR) is a biomarker reflecting the balance between inflammation (as indicated by the neutrophil count) and adaptive immunity (as indicated by the lymphocyte count). We aimed to estimate ability of NLR at admission and at day 1 for predicting stroke outcome after two reperfusion therapies: intravenous thrombolysis (IVT) and mechanical thrombectomy (MT).

**Methods:**

A retrospective analysis was performed on patients who received recombinant human tissue plasminogen activator (IVT) and/or underwent MT for acute ischemic stroke (AIS) at the First Affiliated Hospital of Wenzhou Medical University (Wenzhou, China) from January 2018 to December 2020. Blood samples were taken on admission to hospital and on day 1 after stroke onset. Binary logistic regression models were applied to investigate potential associations between NLR at admission or day 1 and the following outcomes: symptomatic intracerebral hemorrhage (sICH), dependence, and mortality at 90 days. The ability of NLR to predict AIS outcome was analyzed using receiver operating characteristic (ROC) curves.

**Results:**

Data for 927 patients (576 IVT and 351 MT) were reviewed. High admission NLR was associated with dependence in IVT treatment [adjusted odds ratio (OR) 1.21, 95% confidence interval (CI) 1.14–1.23] and 90-day mortality in MT patients (OR 1.09, 95% CI 1.04–1.13). In IVT patients, high NLR at day 1 predicted dependence (OR 1.09, 95% CI 1.02–1.11), sICH (OR = 1.07, 95% CI 1.01–1.12), and 90-day mortality (OR 1.06, 95% CI 1.01–1.15). In MT patients, high NLR at day 1 also predicted dependence (OR 1.08, 95% CI 1.02–1.11) and sICH (OR 1.03, 95% CI 1.01–1.09). ROC analysis confirmed that NLR at day 1 could predict dependence (cut-off 4.2; sensitivity 68.7%; specificity 79.6%), sICH (cut-off 5.1; sensitivity 57.9%, specificity 73.5%), and death (cut-off 5.4; sensitivity 78.8%; specificity 76.4%) in IVT patients. Z values of area under the curves were compared between admissioin and day 1 NLR in IVT patients and showed day 1 NLR can better predict dependence (*Z* = 2.8, *p* = 0.004) and 90-day death (*Z* = 2.8, *p* = 0.005).

**Conclusions:**

NLR is a readily available biomarker that can predict AIS outcome after reperfusion treatment and day 1 NLR is even better than admission NLR.

## Introduction

Reperfusion therapies, namely, intravenous thrombolysis (IVT) and mechanical thrombectomy (MT), are the most effective treatment for patients with acute ischemic stroke (AIS), but they are associated with symptomatic intracerebral hemorrhage (sICH) and ischemic-reperfusion injury. Emerging evidence indicates that post-stroke immune responses can affect the neurovascular interface, leading to reperfusion injury and sICH. Neutrophils are among the first cells in the blood to respond after ischemic stroke, and they contribute to the disruption of the blood–brain barrier (BBB), cerebral edema, and brain injury (1). After ischemic stroke, the number of circulating neutrophils rises while the number of lymphocytes falls, resulting in an increased neutrophil-to-lymphocyte ratio (NLR) (2, 3). Some studies investigated the relationship between admission NLR and prognosis of patients with ischemic stroke treated with IVT or MT (4–7) and found that high pretreatment NLR was associated with sICH or worse 3-month functional outcomes. This may be due to neuroinflammation induced by increased NLR (5, 8). In patients with ischemic stroke, the number of circulating neutrophils begins to rise within 6 h, but neutrophil infiltration into the brain peaks at 24–48 h after stroke onset (9, 10). In addition, the levels of proinflammatory cytokines produced by neutrophils such as tumor necrosis factor-alpha increase within 24–48 h after stroke onset, then fall slightly by 72–144 h (11). The time gap between the early increase in circulating neutrophils and the peak of neuroinflammation begs the question of whether the NLR increase causes subsequent inflammation-induced outcomes. Thus, it seems plausible to speculate that the NLR increase at 24 h after stroke onset may predict stroke outcome better, but the evidence is lacking. If NLR on day 1 has a prognostic value, then it may extend the time window for effective stroke treatment. Thus, in this study, we assessed whether NLR at admission and on day 1 after stroke onset is associated with stroke outcomes after IVT or MT. We then estimated cut-off values of NLR to predict outcomes in patients undergoing IVT or MT.

## Materials and methods

### Study population

We conducted a retrospective, observational study at the First Affiliated Hospital of Wenzhou Medical University (Wenzhou, China) among patients consecutively included in the hospital registry from January 2018 to December 2020. Patients were included in the present study if they were diagnosed with ischemic stroke and were treated with IVT, MT, or both.

**Figure 1:**
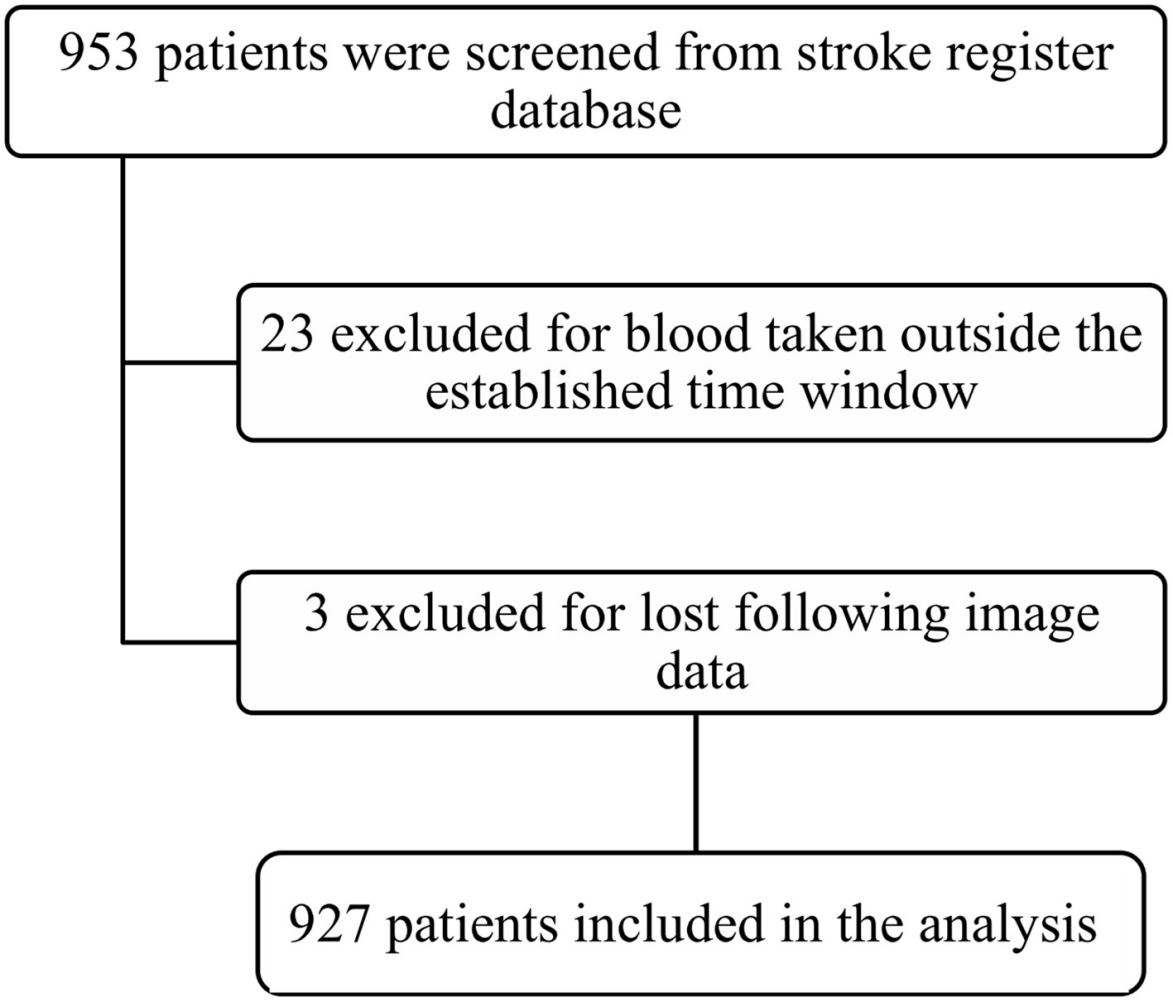
Patient flow-chart of the cohort.

Patients were included whether they had available laboratory values with complete blood count from blood collected at admission (up to 4.5 h before IVT and up to 6 h before MT) and at day 1, corresponding to 12–36 h from stroke onset (referring to time from last known well in case of unwitnessed onset). The collected blood was mixed well and inserted into Sysmex Automated Analyzer by Mindray BC 6800, Shenzhen, China. Total white blood cell counts (WBCs) ×10^9^/L and their differentials neutrophils and lymphocytes were measured. Out of 953 patients with AIS treated with reperfusion, 927 total patients were included in the study, after excluding 23 patients whose blood samples were taken outside the established time window and 3 patients for missing follow-up imaging data (Figure 1). Blood collection was available at admission in 793 (85.5%) patients after a median time of 3.4 (0.7–5.9) h from stroke onset, whereas on day 1 in 924 (99.7%) patients after 22.9 (16.2–29.7) h from stroke onset. Seven hundred ninety patients (85.2%) had both admission and day 1 complete blood counts available.

For each patient, we recorded demographic data, prestroke functional status using a modified Rankin Scale (mRS), and vascular risk factors, namely, hypertension, diabetes mellitus, hyperlipidemia, stroke history, current smoker, and atrial fibrillation. Whether the patient had a history of atrial fibrillation and stroke was determined based on the patient's or the relatives' statement and confirmed by a review of the patient's past medical history. Hypertension and diabetes were assessed according to the 2010 Chinese guidelines for the management of hypertension (12) and the 2018 American Diabetes Association Standards of Medical Care in Diabetes (13), respectively. Hyperlipidemia was diagnosed when one of the following criteria was met: total cholesterol level ≥6.2 mmol/L, low-density lipoprotein cholesterol level ≥4.1 mmol/L, triglyceride level ≥2.3 mmol/L, or high-density lipoprotein chole <1.0 mmol/L. Current smoking is defined as regular smoking of at least 1 cigarette per day at the time of presentation. We recorded stroke severity assessed with the National Institutes of Health Stroke Scale (NIHSS) on admission and on day 1; administration of systemic thrombolysis; the site of the large vessel occlusion on baseline CT angiography; and stroke cause according to TOAST classification (14). Because of the small number for each type of small artery occlusion, the stroke of other determined etiology and undetermined etiology, these three types of stroke are referred to as “other” type together. NLR was calculated as the ratio of the number of neutrophils to the number of lymphocytes. The baseline NIHSS score was determined at admission by a staff neurologist. Treatment was classified as having received IVT [by recombinant human tissue plasminogen activator (rtPA) at the dose of 0.9 mg/kg] or mechanical thrombectomy (MT) whether with or without pretreatment of IVT. The outcome was measured by mRS at 90 days during clinical follow-up by trained staff. Additional demographic information was identified from the medical record.

### Study outcomes

The occurrence of hemorrhagic transformation and sICH, after treatment, was assessed in one or more of the imaging techniques available during 24-h follow-up: non-contrast CT scan in 677 (73%) patients and brain magnetic resonance imaging in 436 (47%) patients. Symptomatic intracerebral hemorrhage was defined according to ECASS-II (European–Australian Acute Stroke Study II) definition (15): any HT and worsening by ≥4 points on the NIHSS. Functional outcomes at 3 months were assessed with the modified Rankin Scale (mRS) in-person or through a telephone interview by a certified evaluator. Dependence or poor outcome were defined as mRS > 3 points. Death within 90 days and sICH were recorded as an index of safety outcomes.

The local Clinical Research Ethics Committee approved the study protocol under the requirements of national legislation in the field of biomedical research, the regulation (EU) 2016/679 of the European Parliament and of the Council of 27 April 2016 on the protection of natural persons with regard to the processing of personal data and on the free movement of such data, and the standards of Good Clinical Practice, and also with the Helsinki Declaration of 1975/1983. Patient consent was not required because of the retrospective nature of the study design and the lack of patient interaction.

### Statistical analyses

Descriptive and frequency analyses were conducted for all demographic and clinical data. The normal distribution of data was tested by skewness and kurtosis analyses. The continuous variables in our study are all non-normal distributions. Changes in neutrophils, lymphocytes, and NLR before and after reperfusion procedures were compared by Wilcoxon signed ranks test. Univariate tests (χ^2^-test for categorical variables, Mann–Whitney *U*-test for continuous variables) were first used to compare clinical, neuroradiological features, and NLR in patients for dependence, death, and sICH. Multivariable logistic regression models were used to test the independent effect of NLR on outcome measures including age and sex and other variables with a *P* < 0.1 in univariate analysis. Receiver operating characteristic (ROC) curves were used to determine the predictive values of the area under the curve and 95% CI. We considered an area under the curve value of 0.70 or higher as indicating acceptable discrimination. To compare the ability of NLR to predict stroke prognosis, we calculated the *Z*-value of the areas under the curve by the formula *Z* = (S1-S2)/(SE1^*^SE1+SE2^*^SE2)^∧^0.5 and compared the area by the *Z*-test. All statistical analyses were performed using IBM SPSS Statistics for Mac, version 23.0 (IBM Corp, Armonk, NY, USA) and the figures are performed using GraphPad Prism 9.0 (GraphPad Software Inc., San Diego, CA, USA).

## Results

### Baseline characteristics and study outcomes

Patients' clinical and demographic baseline characteristics and stroke outcomes are displayed in Table 1. Across all patients, the median baseline NIHSS score was 9 [interquartile range (IQR) (4–14)] and the median 90-day mRS was 2 (IQR 0–4). Among the 351 patients treated with MT, 88 patients (25.1%) received IVT before MT. The median baseline NIHSS score was 14 (IQR 11–19) and the median 90-day mRS was 4 (IQR 1–5). Anterior circulation strokes comprised 85.7% of the population.

**Table 1:** Baseline characteristics of the study population and study outcomes.

**Demographic characteristics**	**Total (*n* = 927)**	**IVT (*n* = 576)**	**MT (*n* = 351)**	***p***
Age (years, median, IQR)	68 (59–76)	68 (59–76)	69 (60–76)	0.88
Sex, male *n* (%)	628 (68)	379 (65)	249 (71)	0.08
Hypertension, *n* (%)	664 (72)	417 (72)	247 (70)	0.59
Diabetes mellitus, *n* (%)	247 (30)	173 (30)	101 (29)	0.72
Hyperlipidemia, *n* (%)	86 (9)	47 (7)	39 (11)	0.2
Atrial fibrillation, *n* (%)	320 (35)	164 (28)	156 (44)	<0.001^*^
Current smoker, *n* (%)	152 (16)	145 (12)	7 (6)	0.067
Stroke history, *n* (%)	93 (10)	38 (7)	55 (16)	<0.001^*^
Admission NIHSS score, median (IQR)	9 (4–14)	5 (3–10)	14 (11–19)	<0.001^*^
mRS 0–1 before stroke, *n* (%)	859 (93)	549 (95)	310 (88)	<0.001^*^
Stroke onset to treat time, min, median (IQR)	249 (196–307)	173 (126–221)	318 (278–352)	<0.001^*^
Stroke etiology (TOAST) *n* (%)				
Large-artery atherosclerosis	557 (60)	373 (65)	184 (52)	<0.001^*^
Cardioembolism	284 (31)	128 (22)	156 (44)	<0.001^*^
Other	88 (9)	77 (13)	11 (3)	<0.001^*^
Anterior circulation, *n* (%)	794 (86)	497 (86)	297 (85)	0.56
Day 1 NIHSS score, median (IQR)	6 (2–13)	3 (1–8)	11 (7–21)	<0.001^*^
Hemorrhagic transformation, *n* (%)	172 (19)	67 (12)	105 (30)	<0.001^*^
sICH, *n* (%)	82 (9)	43 (8)	39 (11)	0.05
Functional outcome dependence (mRS>3), *n* (%)	376 (410)	143 (25)	233 (66)	<0.001^*^
mRS score at 90 day, median (IQR)	2 (0–4)	1 (0–2)	4 (1–5)	<0.001^*^
Mortality, *n* (%)	116 (13)	36 (6)	80 (23)	<0.001^*^
Admission WBC, median (IQR)	7.6 (6.0–9.7)	7.2 (5.8–9.1)	8.3 (6.4–10.7)	<0.001^*^
Admission neutrophils, median (IQR)	5.0 (3.6–7.1)	4.6 (3.5–6.4)	5.9 (4.2–8.6)	<0.001^*^
Admission lymphocytes, median (IQR)	1.6 (1.2–2.1)	1.7 (1.3–2.2)	1.5 (1.1–1.9)	<0.001^*^
Admission NLR, median (IQR)	3.7 (1.7–6.8)	2.6 (1.7–4.3)	4.3 (2.5–6.8)	<0.001^*^
Day 1 WBC, median (IQR)	8.3 (6.4–11.7)	7.6 (6.4–9.6)	9.6 (7.6–11.7)	<0.001^*^
Day 1 neutrophils, median (IQR)	6.1 (4.8–8.1)	5.4 (4.8–6.1)	7.7 (7.1–8.1)	<0.001^*^
Day 1 lymphocytes, median (IQR)	1.4 (1.0–2.1)	1.5 (1.1–2.1)	1.3 (1.0–1.8)	<0.001^*^
Day 1 NLR, median (IQR)	4.3 (2.7–8.6)	3.5 (2.7–5.6)	5.9 (3.9–8.6)	<0.001^*^

*IVT, intravenous thrombolysis; MT, mechanical thrombectomy; NIHSS, National Institutes of Health Stroke Scale; IQR, interquartile range; TOAST, trial of Org 10 172 in acute stroke treatment; WBC, while blood cell; sICH, symptomatic intracerebral hemorrhagic; NLR, neutrophil over lymphocytes ratio.*

* *Statistically significant*.

On admission, the median neutrophil percentage was 68% (IQR 57–78%) of the total WBC and the median lymphocyte percentage was 23% (IQR 14–31%), corresponding to a median NLR of 3.7 (IQR 1.7–6.8). On day 1, the median neutrophil percentage was 75% (IQR 66–83%) and the median lymphocyte percentage was 17% (IQR 12–25%), corresponding to a median NLR of 4.3 (IQR 2.7–8.6).

Across the entire cohort, 82 (8.8%) patients presented sICH and 116 (12.5%) died. A bad outcome was presented in 376 (39.6%) presented bad outcomes on day 90.

Patients with MT had significant more previous stroke history (15.7 vs. 6.6%), previous disability (11.7 vs. 4.7%), sICH after reperfusion therapy (11.1 vs. 7.5%), and more functional dependence at 90 days (66.4 vs. 24.8%) compared to the IVT group. Recanalization, which is demonstrated by a myocardial infarction (TIMI) score of 2 or 3, was achieved in 260 (74.1%) patients who underwent MT.

### Changes in neutrophils, lymphocytes, and NLR before and after reperfusion procedures

**Figure 2:**
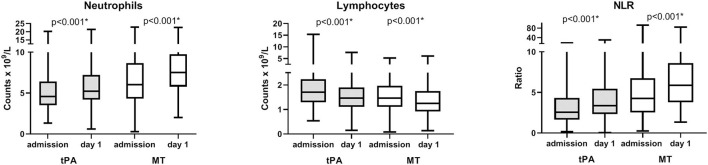
Longitudinal changes of neutrophil, lymphocyte counts, and neutrophil-to-lymphocyte ratio (NLR) over time. Analysis of repeated measures of neutrophil, lymphocyte counts, and NLR at admission and at day 1 in patients with both admission and day 1 complete blood counts available (*n* = 495 in tPA and *n* = 295 in MT). *P*-value refer to comparisons of repeated measures. Boxes, 25–75% interquartile range; central horizontal bars, median; outer horizontal bars, minimum and maximum values. Significant (*P* < 0.001) differences between admission and day 1 values were also obtained in comparisons of means (Mann–Whitney *U*-test) on the entire population (data not shown). IVT, intravenous thrombolysis; MT, mechanical thrombectomy.

In 790 patients with both admission and day 1, complete blood counts were available (*n* = 495 in IVT and *n* = 295 in MT), neutrophils increase in 516 (65.3%), lymphocytes decrease in 517 (65.4%), and NLR increased in 520 (65.8%) patients at day 1 compared to admission. In both patients with IVT and MT, neutrophils and NLR increase and lymphocytes decrease on day 1 (all *p* < 0.001, Figure 2).

### Compare baseline characteristics and NLR in patients presented with dependence or not

In both patients with IVT and MT, high admission NIHSS is related to poor outcomes (both *p* < 0.001, Table 2) and old age is another factor leading to dependence (*p* < 0.001 and *p* = 0.002, respectively, Table 2). Female, atrial fibrillation, high admission, longer stroke onset to puncture time, and day 1 NLR in patients with IVT predict worse functional outcomes (*p* = 0.04, *p* < 0.0001, *p* < 0.0001, *p* = 0.03, and *p* < 0.0001, respectively, Table 2). Stroke classification types are different in the IVT group (Table 2). High day 1 NLR is associated the poor outcome in patients with MT (*p* = 0.01, Table 2).

**Table 2:** Comparison of clinical characteristics and NLR in patients presented with dependence or not at 90 day.

	**IVT**				**MT**			
	**Yes (*n* = 108)**	**No (*n* = 468)**	**Statistic value**	***p***	**Yes (*n* = 187)**	**No (*n* = 164)**	**Statistic value**	***p***
Admission NIHSS, median (IQR)	12 (9–15)	4 (2–8)	−12.8	<0.001^*^	16 (12–23)	12 (10–16)	−6.4	<0.001^*^
Age, median (IQR)	76 (68–83)	67 (58–74)	−7.2	<0.001^*^	71 (61–78)	66 (59–74)	−3.1	0.002^*^
Male, *n* (%)	62 (57)	317 (67)	4.2	0.04^*^	125 (66)	124 (75)	3.3	0.07
Hypertension, *n* (%)	79 (73)	338 (72)	0.04	0.85	134 (71)	113 (68)	0.32	0.57
Diabetes mellitus, *n* (%)	33 (30)	140 (30)	0.02	0.9	60 (32)	41 (25)	2.1	0.14
Hyperlipidemia, *n* (%)	5 (4)	42 (9)	2.2	0.14	16 (8)	23 (14)	2.6	0.1
Current smoker, *n* (%)	36 (33)	109 (23)	4.7	0.03	2 (1)	5 (3)	1.8	0.2
Stroke history, *n* (%)	11 (10)	27 (6)	2.8	0.09	34 (18)	21 (13)	1.9	0.2
mRS 0–1 before stroke, *n* (%)	101 (94)	448 (96)	0.9	0.3	169 (90)	141 (86)	1.6	0.2
Atrial fibrillation, *n* (%)	46 (42)	118 (22)	13.0	<0.001^*^	92 (49)	64 (39)	3.7	0.06
Admission NLR, median (IQR)	4.1 (2.5–6.1)	2.4 (1.6–4.0)	−4.8	<0.001^*^	4.5 (2.7–6.8)	3.9 (2.4–6.8)	−1.5	0.13
Day 1 NLR, median (IQR)	6.2 (4.4–10)	3.1 (2.2–4.9)	−8.9	<0.001^*^	6.4 (4.1–9.3)	5.2 (3.6–7.9)	−2.5	0.01^*^
Day 1 NHISS, median (IQR)	13 (10–20)	3 (1–5)	−13.9	<0.001^*^	12 (7–22)	7 (3–10)	−14.1	<0.001^*^
Stroke onset to treat time, min, median (IQR)	166 (127–188)	163 (122–185)	−1.2	0.16	314 (277–353)	321 (271–358)	−1.3	0.18
Stroke etiology (TOAST), *n* (%)								
Large-artery atherosclerosis	63 (58)	310 (66)	2.4	0.1	98 (52)	86 (52)	0	0.99
Cardioembolism	16 (15)	112 (24)	4.2	0.04^*^	80 (43)	76 (46)	0.5	0.5
Other	29 (27)	48 (10)	20.1	<0.001^*^	9 (5)	2 (2)	3.7	0.05
Anterior circulation, *n* (%)	93 (86)	404 (86)	0.3	0.6	157 (84)	140 (85)	0.1	0.7

*IVT, intravenous thrombolysis; MT, mechanical thrombectomy; NIHSS, National Institutes of Health Stroke Scale; IQR, interquartile range; NLR, neutrophil over lymphocytes ratio.*

* *Statistically significant*.

### Compare baseline characteristics and NLR in patients presented with sICH or not

In IVT, high admission NIHSS and old age are related to sICH (*p* < 0.001 and *p* = 0.001, Table 3). Female, disability before the stroke and atrial fibrillation are associated with sICH (*p* = 0.03, *p* < 0.001, and *p* = 0.002, respectively, Table 3). Admission and day 1 NLR is significantly higher in patients with sICH than in no sICH ones (*p* = 0.001 and *p* < 0.001, Table 3). In patients with MT, DM predicts sICH (*p* = 0.03, Table 3). No differences in either admission or day 1 NLR are found between patients with sICH and no sICH in MT.

**Table 3:** Comparison of clinical characteristics and NLR in patients presented with sICH or not.

	**IVT**				**MT**			
	**Yes (*n* = 43)**	**No (*n* = 533)**	**Statistic value**	***p***	**Yes (*n* = 39)**	**No (*n* = 312)**	**Statistic value**	***p***
Admission NIHSS, median (IQR)	13 (8–17)	5 (2–10)	−6.5	<0.001^*^	16 (11–22)	14 (11–19)	−1.51	0.13
Age, median (IQR)	75 (67–83)	68 (58–76)	−3.4	0.001^*^	70 (59–78)	69 (60–76)	−0.39	0.69
Male, *n* (%)	22 (51)	361 (68)	4.9	0.03^*^	28 (72)	203 (74)	0.69	0.4
Hypertension, *n* (%)	31 (72)	376 (71)	0.05	0.83	29 (74)	218 (70)	0.34	0.56
Diabetes mellitus, *n* (%)	16 (37)	157 (29)	1.14	0.3	17 (44)	84 (27)	4.6	0.03^*^
Hyperlipidemia, *n* (%)	1 (2)	46 (9)	2.1	0.15	8 (21)	31 (10)	3.9	0.05
Current smoker, *n* (%)	9 (20)	136 (26)	0.4	0.5	1 (3)	6 (2)	0	1
Stroke history, *n* (%)	2 (5)	36 (7)	0.3	0.6	6 (15)	49 (16)	0.003	0.96
mRS 0–1 before stroke, *n* (%)	33 (77)	507 (95)	22.9	<0.001^*^	35 (91)	275 (88)	0.001	0.98
Atrial fibrillation, *n* (%)	21 (49)	143 (27)	9.6	0.002^*^	20 (51)	136 (44)	0.83	0.36
Admission NLR, median (IQR)	4.2 (2.2–5.9)	2.5 (1.6–4.2)	−3.2	0.001^*^	3.6 (2.2–6.0)	4.3 (2.5–6.8)	−0.97	0.33
Day 1 NLR, median (IQR)	7.1 (3.9–10.6)	3.3 (2.3–5.3)	−5.3	<0.001^*^	6.1 (4.1–9.4)	5.8 (3.8–8.6)	−0.73	0.47
Day 1 NHISS, median (IQR)	16 (9–26)	3 (1–7)	−8.1	<0.001^*^	21 (12–29)	11 (6–18)	−4.1	<0.001^*^
Stroke onset to treat time, min, median (IQR)	164 (122–182)	167 (123–185)	−1.1	0.2	315 (277–352)	318 (270–356)	−1.3	0.2
Stroke etiology (TOAST), *n* (%)								
Large-artery atherosclerosis	29 (67)	343 (64)	1.1	0.3	20 (51)	164 (53)	0.02	0.9
Cardioembolism	11 (26)	117 (22)	0.3	0.6	17 (43)	139 (45)	0.01	0.9
Other	4 (9)	73 (14)	0.07	0.4	2 (5)	9 (3)	0.07	0.8
Anterior circulation, *n* (%)	35 (82)	462 (87)	0.94	0.3	38 (97)	259 (83)	5.5	0.01

*IVT, intravenous thrombolysis; MT, mechanical thrombectomy; NIHSS, National Institutes of Health Stroke Scale; IQR, interquartile range; NLR, neutrophil over lymphocytes ratio.*

* *Statistically significant*.

### Compare baseline characteristics and NLR in patients presented with death or not

In patients with IVT, high admission NIHSS, female, and old age are related to 90-day death (*p* < 0.001, *p* < 0.001, and *p* = 0.02, respectively, Table 4). Atrial fibrillation and high day 1 NLR were associated with death (*p* = 0.01 and *p* < 0.001, Table 4). In patients with MT, high admission NIHSS and old age are related to 90-day death (*p* = 0.007 and *p* < 0.001, Table 4). DM and high day 1 NLR are found to relate to 90-day death (both *p* = 0.03, Table 4).

**Table 4:** Comparison of clinical characteristics and NLR in patients presented 90 death or not.

	**IVT**				**MT**			
	**Yes (*n* = 34)**	**No (*n* = 542)**	**Statistic value**	***p***	**Yes (*n* = 80)**	**No (*n* = 271)**	**Statistic value**	***p***
Admission NIHSS, median (IQR)	15 (12–17)	5 (2–10)	−10.5	<0.001^*^	19 (12–24)	14 (10–17)	−2.7	0.007^*^
Age, median (IQR)	81 (72–88)	68 (58–76)	−5.5	<0.001^*^	72 (63–79)	68 (59–76)	−5.0	<0.001^*^
Male, *n* (%)	16 (47)	363 (67)	5.6	0.02^*^	59 (73)	190 (70)	0.4	0.5
Hypertension, *n* (%)	25 (73)	392 (72)	0.02	0.9	57 (71)	190 (70)	0.04	0.84
Diabetes mellitus, *n* (%)	11 (32)	162 (29)	0.1	0.8	31 (38)	70 (25)	5	0.03^*^
Hyperlipidemia, *n* (%)	6 (18)	41 (8)	0.6	0.4	12 (15)	27 (10)	2.3	0.1
Atrial fibrillation, *n* (%)	16 (47)	148 (27)	6.1	0.01^*^	31 (38)	125 (46)	1.4	0.4
Current smoker, *n* (%)	9 (26)	136 (25)	0.03	0.8	1 (1)	6 (2)	0.3	0.6
Stroke history, *n* (%)	2 (5)	36 (7)	0.03	0.8	11 (14)	44 (16)	0.3	0.6
mRS 0–1 before stroke, *n* (%)	31 (91)	518 (95)	1.3	0.2	72 (90)	238 (88)	0.3	0.6
Admission NLR, median (IQR)	3.8 (1.9–5.7)	2.5 (1.6–4.3)	−1.7	0.09	4.5 (2.7–8.9)	4.2 (2.5–6.4)	−1.7	0.09
Day 1 NLR, median (IQR)	8.6 (5.5–12.9)	3.3 (2.3–5.4)	−6	<0.001^*^	7.1 (4.1–10.1)	5.5 (3.8–8.4)	−2.2	0.03^*^
Day 1 NHISS, median (IQR)	26 (19–30)	3 (1–7)	−9.4	<0.001^*^	29 (27–32)	10 (5–14)	−13.2	<0.001^*^
Stroke onset to treat time, min, median (IQR)	167 (122–179)	162 (119–183)	−1.2	0.16	323 (267–364)	319 (271–347)	−1.1	0.21
Stroke etiology (TOAST), *n* (%)								
Large-artery atherosclerosis	24 (71)	349 (64)	0.5	0.5	37 (46)	147 (54)	0.2	0.7
Cardioembolism	7 (20)	121 (22)	0.05	0.8	35 (44)	121 (45)	0.1	0.6
Other	5 (15)	70 (13)	0.02	0.9	8 (10)	3 (1)	0.002	0.9
Anterior circulation, *n* (%)	27 (79)	470 (87)	1.4	0.2	71 (89)	226 (83)	1.4	0.2

*IVT, intravenous thrombolysis; MT, mechanical thrombectomy; NIHSS, National Institutes of Health Stroke Scale; IQR, interquartile range; NLR, neutrophil over lymphocytes ratio.*

* *Statistically significant*.

### Predictive values of NLR for three end points

**Figure 3:**
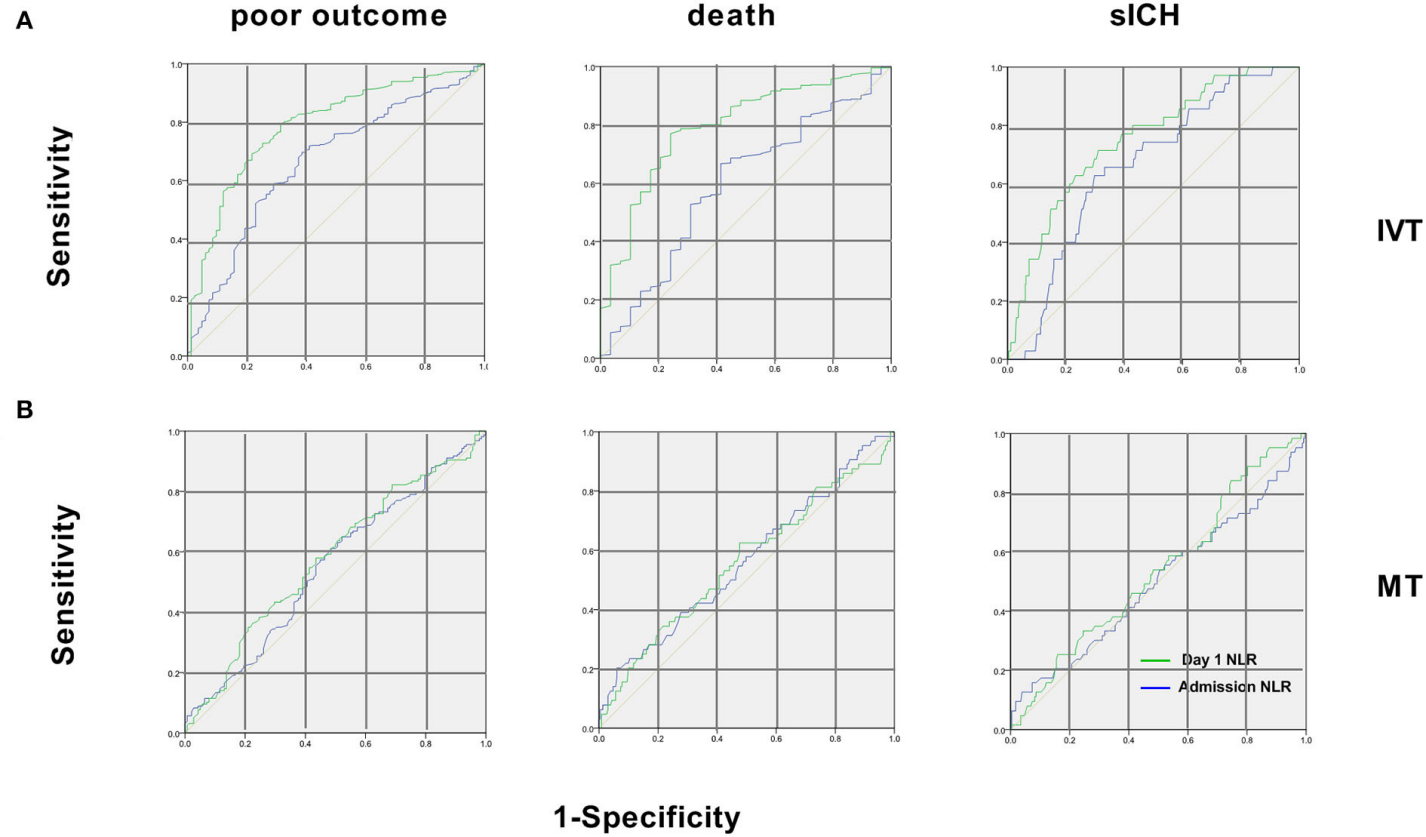
Receiver operating characteristic curves of NLR for the 3 endpoints in patients with IVT and MT. **(A)** In patients with IVT, for poor outcome, areas under the curve were 0.66 for admission NLR and 0.78 for day 1 NLR. For 90-day death, areas were 0.60 for admission NLR and 0.81 for day 1 NLR. For sICH, areas were 0.63 for admission NLR and 0.74 for day 1 NLR; **(B)** In patients with MT, areas under the curve were 0.55 (95% CI 0.48–0.6) for admission NLR and 0.57 (95% CI 0.48–0.66) for day 1 NLR for poor outcome. For 90-day death, areas were 0.57 (95% CI 0.44–0.63) for admission NLR and 0.58 (95% CI 0.45–0.67) of day 1 NLR. For sICH, areas were 0.51 (95% CI 0.42–0.65) for admission NLR and 0.56 (95% CI 0.43–0.6) for day 1 NLR. IVT, intravenous thrombolysis; MT, mechanical thrombectomy; sICH, symptomatic intracerebral hemorrhage.

The ROC curves and the area underneath them for using NLR to predict outcomes are shown in Figure 3. For predicting poor outcomes in patients with IVT generally, NLR on day 1 showed a sensitivity of 68.7%, specificity of 79.6%, a positive likelihood ratio 2.33, and an area under the curve of 0.79 (95% CI 0.73–0.82, *p* < 0.001). For predicting death in patients with IVT, NLR on day 1 showed a sensitivity of 78.8%, specificity of 76.4%, a positive likelihood ratio of 1.03, and an area under the curve of 0.81 (95% CI 0.74–0.89, *p* < 0.001). For predicting sICH in patients with IVT, NLR on day 1 showed a sensitivity of 71.4%, specificity of 68.9%; a positive likelihood ratio of 2.11, and an area under the curve of 0.74 (95% CI 0.67–0.82, *p* < 0.001). The cut-off NLR of dependence, sICH, and death were 4.2, 5.1, and 4.7, respectively.

While for the NLR at admission for patients with IVT and that of patients with MT at admission and on day 1, the areas of ROC are under the discrimination value (Figure 3).

To further assess the ability of NLR to predict prognosis, *Z* values of the areas under ROC curves were compared between NLR at admission and day 1 in patients with IVT. We found that high NLR on day 1 predicted poor outcome and death at 90 days better than NLR at admission, while the two NLRs were similar in their ability to predict sICH (Table 5).

**Table 5:** Comparison of the area under ROC curve between admission and day 1 NLR in patients with IVT.

**Area under the curve**	**Dependence (95% CI)**	**Death (95% CI)**	**sICH (95% CI)**
Admission	0.66 (0.60–0.73)	0.60 (0.49–0.70)	0.66 (0.58–0.74)
Day 1	0.78 (0.73–0.82)	0.80 (0.74–0.89)	0.75 (0.63–0.83)
*Z*-value	2.8	2.8	1.7
*p*	0.004^*^	0.005^*^	0.08

*NLR, neutrophil over lymphocytes ratio; IVT, intravenous thrombolysis; sICH, symptomatic intracerebral hemorrhage.*

* *Statistically significant*.

### Association of NLR to three end points before and after adjustment

Before adjustment, in patients with IVT, both admission and day 1 NLR related to patients with poor outcomes at 3 months (*p* < 0.001, Table 6) while only high day 1 NLR is associated with sICH and death within 3 months (both *p* < 0.001, Table 6). In patients with MT, admission NLR predicts death at 90 days (*p* = 0.03, Table 6) and on day 1 NLR predicts worse outcome and sICH (*p* = 0.03 and *p* = 0.02, Table 6) before adjustment.

**Table 6:** Unadjusted multivariate analysis for neutrophil–lymphocyte ratio (NLR) prediction models.

	**IVT**				**MT**			
	**Admission**		**Day 1**		**Admission**		**Day 1**	
	**OR (95% CI)**	***p***	**OR (95% CI)**	***p***	**OR (95% CI)**	***p***	**OR (95% CI)**	***p***
Poor outcome	1.21 (1.14–1.23)	<0.001^*^	1.21 (1.09–1.24)	<0.001^*^	0.98 (0.96–1.12)	0.07	1.01 (1.01–1.07)	0.03^*^
sICH	1.07 (0.89–1.12)	0.95	1.16 (1.08–1.24)	<0.001^*^	1.01 (0.98–1.05)	0.31	1.05 (1.01–1.10)	0.02^*^
Death	1.07 (0.84–1.32)	0.9	1.22 (1.07–1.24)	<0.001^*^	0.88 (0.83–1.01)	0.03^*^	0.88 (0.86–1.01)	0.05

*IVT, intravenous thrombolysis; MT, mechanical thrombectomy; sICH, symptomatic intracerebral hemorrhagic.*

* *Statistically significant*.

After adjustment, admission NLR is still related to the poor outcome at 90 days (*p* < 0.001, Table 7) and on day 1 NLR is associated with poor outcome, sICH, and mortality (*p* = 0.002, *p* = 0.04, and *p* = 0.003, Table 7) in patients with IVT. For patients with MT, after adjustment, admission NLR is still related to 90-day mortality (*p* = 0.04, Table 7) and day 1 NLR is associated with poor outcome and sICH (both *p* = 0.04, Table 7).

**Table 7:** Adjusted multivariate analysis for neutrophil–lymphocyte ratio (NLR) prediction models.

	**IVT**				**MT**			
	**Admission**		**Day 1**		**Admission**		**Day 1**	
	**OR (95% CI)**	***p***	**OR (95% CI)**	***p***	**OR (95% CI)**	***p***	**OR (95% CI)**	***p***
Poor outcome	1.11 (1.07–1.16)	<0.001^*^	1.09 (1.02–1.11)	0.002^*^	1.13 (0.99–1.11)	0.09	1.08 (1.02–1.11)	0.04^*^
sICH	1.03 (0.93–1.15)	0.5	1.06 (1.01–1.13)	0.04^*^	1.01 (0.98–1.04)	0.47	1.05 (1.01–1.10)	0.04^*^
Death	0.88 (0.64–1.12)	0.3	1.06 (1.01–1.15)	0.003^*^	1.09 (1.04–1.13)	0.04^*^	0.98 (0.96–1.08)	0.07

*IVT, intravenous thrombolysis; MT, mechanical thrombectomy; sICH, symptomatic intracerebral hemorrhagic.*

* *Statistically significant. The associations were adjusted for admission NIHSS score, age, sex, history of diabetes and atrial fibrillation, prestroke disability, day 1 NIHSS score, and stroke type*.

## Discussion

Here we assessed the clinical value of NLR for predicting stroke outcomes after reperfusion therapies. We observed that NLR at admission was associated with dependence and mortality, while NLR on day 1 predicted sICH, dependence, and mortality in patients with IVT at day 90. In patients with MT, admission NLR was associated with mortality, while NLR on day 1 predicted sICH and poor outcomes at day 90. Also, we found that on day 1 was a better prognostic indicator than NLR at admission in patients with AIS who received reperfusion treatment.

Here we confirmed an increase in NLR with time after stroke, which is in consistent with previous studies in patients who undergo IVT or MT (4, 6, 16). After ischemic stroke, complex networks connecting the brain and the immune system are triggered. Within several minutes after stroke, peripheral immune cells are activated and recruited to ischemic tissue, where they exert either beneficial or detrimental effects, depending on the stroke phase and the subtype of leukocytes involved (2, 17, 18). The rise in neutrophils after stroke reflects their enhanced production and release from the bone marrow and spleen, and potentially also reduced apoptosis in neutrophils (19).

In our work, high NLR was associated with an increased risk of sICH after IVT treatment and the findings are consistent with prior studies (6, 16). Circulating neutrophils are recruited to the site of cerebral injury shortly after ischemia occurs, and they contribute to BBB disruption and tissue damage by releasing matrix metalloproteinases, elastase, cathepsin G, proteinase 3, and reactive oxygen species (ROS), while migrating across the cerebral endothelium (20–23). In patients with MT, we observed that NLR at day 1 was related to sICH and poor functional outcomes, also in line with a previous study (4). In other words, NLR on day 1 but not at admission was related to sICH in patients receiving reperfusion treatment. This result indicates a link between NLR at 24 h after stroke and the concurrent peak of neuroinflammation within the brain, which leads to BBB leakage.

Poor outcomes at day 90 were associated with high NLR on day 1 in both reperfusion groups, which may have multiple explanations. First, high NLR at day 1 is associated with sICH, which increases mortality and worsens neurological deficit. Second, except for sICH caused by multifaceted inflammatory response inside the brain parenchymal, cerebral edema, and brain injury which play an important role in functional deterioration, are mediated by factors released from neutrophils including ROS, proteases, cytokines, and chemokines (2). Third, it was reported that neutrophils are also involved in the major processes that cause ischemic stroke, thrombosis, and atherosclerosis (24–26). They promote clot formation through interactions with platelets and release of prothrombotic molecules which may weaken the reperfusion effect or even infarct expansion.

Stroke causes high mortality worldwide, and changes in NLR may affect the mortality rate after stroke. On one hand, For example, high NLR may reflect neutrophil-mediated neuroinflammation as a complication of reperfusion treatment or brain edema that often occurs after a stroke. At the same time, infections such as pneumonia can increase long-term mortality after stroke (27–29). Immunosuppression indicated by lymphocyte decrease following a stroke can increase the risk of such infection. One of the vital mechanisms underlying the post-stroke infection is the impairment of the brain's immune system after a stroke, leading to a stroke-related immunosuppressive syndrome (30, 31). Neutrophils de-differentiate and undergo stimulation by growth factors, while apoptosis reduces lymphocyte numbers, increasing susceptibility to infections (32, 33).

In our study, NLR on day 1 was a better indicator of stroke outcomes in patients with AIS than NLR at admission, which may have several explanations. First, there are interactions between inflammatory agents and neutrophils. Circulating neutrophils recruited to the site of the cerebral facilitate releasing of inflammatory molecules inside the brain. A number of factors released after brain ischemia act on neutrophils including cytokines, chemokines, and damage-associated molecular patterns (DAMPs). These upregulated inflammatory molecules, in turn, further activate neutrophils and provoke additional recruitment of leukocytes from the peripheral blood. Levels of most inflammatory factors peak at 12–72 h after stroke onset, which means that neutrophils are continuously activated (34). Increased neutrophil counts provide a measure of the inflammation in the ischemic brain and they may also contribute directly to neuroinflammation. Second, reperfusion treatment may lead to ischemia-reperfusion injury after focal brain ischemia. Systemic inflammatory responses help circulating neutrophils gain access to the ischemic area and further activate ROS. However, except for increased susceptibility to infections, the mechanism of decreased lymphocyte numbers leading to the worse outcome of AIS remains unclear.

The importance of our work is that we found that posttreatment NLR was a better predictor of stroke outcomes than pre-reperfusion one, suggesting that the strategies to reduce NLR, especially reducing neutrophils after reperfusion is feasible because of extending the time window for treatment and clinically importance. The achievements in the treatment of stroke targeting neutrophils are fruitful in the lab but have challenges to translate to patients (2). The timing and duration of antineutrophil treatment are important determinants of success.

Our work presents several limitations. First, the modest sample size and retrospective analysis of prospectively collected data are important methodological shortcomings. Second chronic inflammatory conditions, prestroke infections, and cancer treatments may all affect a patient's NLR and will need to be taken into account. Intercurrent complications such as infection which is common in severe stroke leading to an increase in neutrophil and NLR cannot be ruled out. It was documented that increased NLR and lymphopenia with or without neutrophilia are linked to stroke-associated pneumonia (35). Third, the skewed distribution of NLR may account for the discrepant findings between Mann–Whitney *U*-test and univariable logistic regression analyses evaluating the unadjusted association of NLR with 90-day three outcome mediators.

## Conclusions

The NLR, particularly on day 1 is a readily available prognosis indicator for AIS outcome after reperfusion treatment.

## Data availability statement

The raw data supporting the conclusions of this article will be made available by the authors, without undue reservation.

## Ethics statement

The studies involving human participants were reviewed and approved by Wenzhou Medical University Affiliated the First Hospital Clinical Research Ethics Committee. Written informed consent for participation was not required for this study in accordance with the national legislation and the institutional requirements.

## Author contributions

SYC, JHC, QY, ZSY, and YLZ searched, reviewed available studies, extracted and analyzed the data, and wrote the paper. YTL, GQH, FCC, MY, CLW, TTD, and XL made critical revisions to the paper. XL extracted the data and co-wrote the paper. ZZ reviewed and made critical revisions to the paper. All authors contributed to the article and approved the submitted version.

## Funding

This work was supported by the Wenzhou Municipal Science and Technology Bureau (Grant Number: Y20180132).

## Conflict of interest

The authors declare that the research was conducted in the absence of any commercial or financial relationships that could be construed as a potential conflict of interest.

## Publisher's note

All claims expressed in this article are solely those of the authors and do not necessarily represent those of their affiliated organizations, or those of the publisher, the editors and the reviewers. Any product that may be evaluated in this article, or claim that may be made by its manufacturer, is not guaranteed or endorsed by the publisher.
